# When expertise became just another opinion: Rebuilding trust through a transparent infrastructure for verified scientific knowledge

**DOI:** 10.1038/s44319-026-00899-x

**Published:** 2026-08-12

**Authors:** Marek Marzec

**Affiliations:** 1 University of Silesia, Research Centre for New Genomic Techniques, Katowice, Poland; 2 University of Silesia, Faculty of Natural Sciences, Katowice, Poland

**Keywords:** History & Philosophy of Science, Methods & Resources, Science Policy & Publishing

## Abstract

Scientific knowledge is advancing faster than ever, yet its authority is increasingly detached from the way information circulates in public debates. This article proposes a transparent, professionally curated repository of validated scientific and educational materials as shared infrastructure for science communication.

**Figure:**
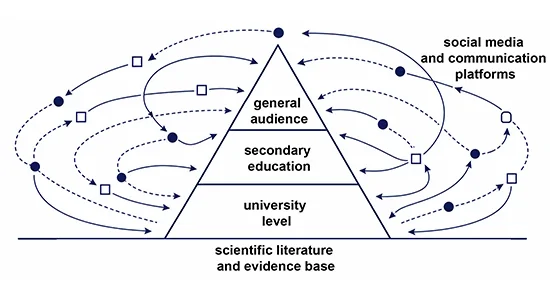

## Graphical abstract

**Figure:**
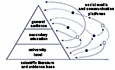

We live in times when the precision of research methods is breaking barriers that, until recently, seemed impossible to cross. We have tools that allow us to monitor the activity of genes or metabolism in individual cells (Yu et al, 2023), we can observe structures as small as 1 nm (Mezache and Leterrier, 2025), and we can precisely replace single nucleotides in the genome (Wang et al, 2024). However, the wider public is often not aware of these tools and the knowledge which helped to design them or the new knowledge they generate. And if laypeople do become interested, the information is not easily accessible to them. The texts that describe scientific results are increasingly difficult to read for non-specialists, published in articles behind paywalls and addressed to a narrow group of experts. This creates an increasing communication gap between rapidly developing science and society.

Moreover, owing to changes in education systems with a shift away from acquiring encyclopaedic knowledge to developing communication and information-acquiring skills, and the rapid rise of social media, many people are exposed to easily accessible, attractively presented, but often false information about, among other things, basic biological facts. Social media algorithms do not care about facts or truths, only about clicks—and the most efficient method to get clicks is to promote polarising information, information that evokes extreme emotions or promises to reveal hidden secrets (McLoughlin et al, 2024). This combination of a lack of basic knowledge and exposure to a large amount of often misleading or outright false information makes society vulnerable to all forms of manipulation, while the moderate voice of scientists is no longer heard in the public debate. The credibility of information is no longer determined by scientific evidence or expert analysis, but by controversy and visibility driven by algorithms.

The credibility of information is no longer determined by scientific evidence or expert analysis, but by controversy and visibility driven by algorithms.

## Case study: new genomic techniques

New genomic techniques (NGTs) are a useful example of how the discourse on online media affects the credibility of science. NGTs enable precise changes in the genome, including single-nucleotide changes, to edit a genome in a way that cannot be distinguished from mutations generated by chemical mutagenesis or natural mutagenesis (Pacher and Puchta, 2017). In some countries, organisms obtained using NGTs are therefore not subject to the legal restrictions that apply to genetically modified organisms (GMOs) (Ricroch et al, 2026). In the EU, Regulation (EU) 2026/1388 on plants obtained by certain NGTs was adopted on 17 June 2026, and most of its provisions will apply from 17 July 2028. The framework reflects scientific assessments indicating that plants produced by targeted mutagenesis and cisgenesis do not inherently present greater risks than plants obtained through conventional breeding, although assessment requirements depend on the type of modification (EFSA, 2020; EP and CEU, 2026). In this context, the NGT Research Centre of the University of Silesia in Katowice, Poland, launched an educational campaign on NGTs, including a coordinated series of social-media posts. The immediate rationale was the growing public and political debate surrounding the proposed EU regulation, in which the distinctions between targeted mutagenesis, transgenesis and conventional breeding were frequently blurred. The campaign consisted of short, visually accessible materials prepared and reviewed by researchers from the Centre. They explained what NGTs are and how they work, clarified how they differ from conventional genetic modification, and presented concrete examples of how these techniques can be used to improve crop plants, for instance by increasing resistance to diseases and environmental stress, enhancing product quality, or modifying agronomically important traits.

It turned out to be a cruel experience of deliberate misunderstanding and misinformation. The fact that some readers or commentators would treat NGTs as just another version of GMOs, regardless of technological and regulatory differences, was to be expected. To reach out to those who would not be ideologically entrenched, we began the campaign by explaining how NGTs work. And this, seemingly logical approach, turned out to be completely ineffective. Leave aside ideologically driven anti-GMO attitudes and the unfortunate trend on social media to escalate discussions into insults— from my point of view, the most disturbing fact was the lack of any differentiation between an argument made by an anonymous participant of the dispute and an expert comment by scientific or governmental institutions supported by scientific evidence.

**Fig. 1:**
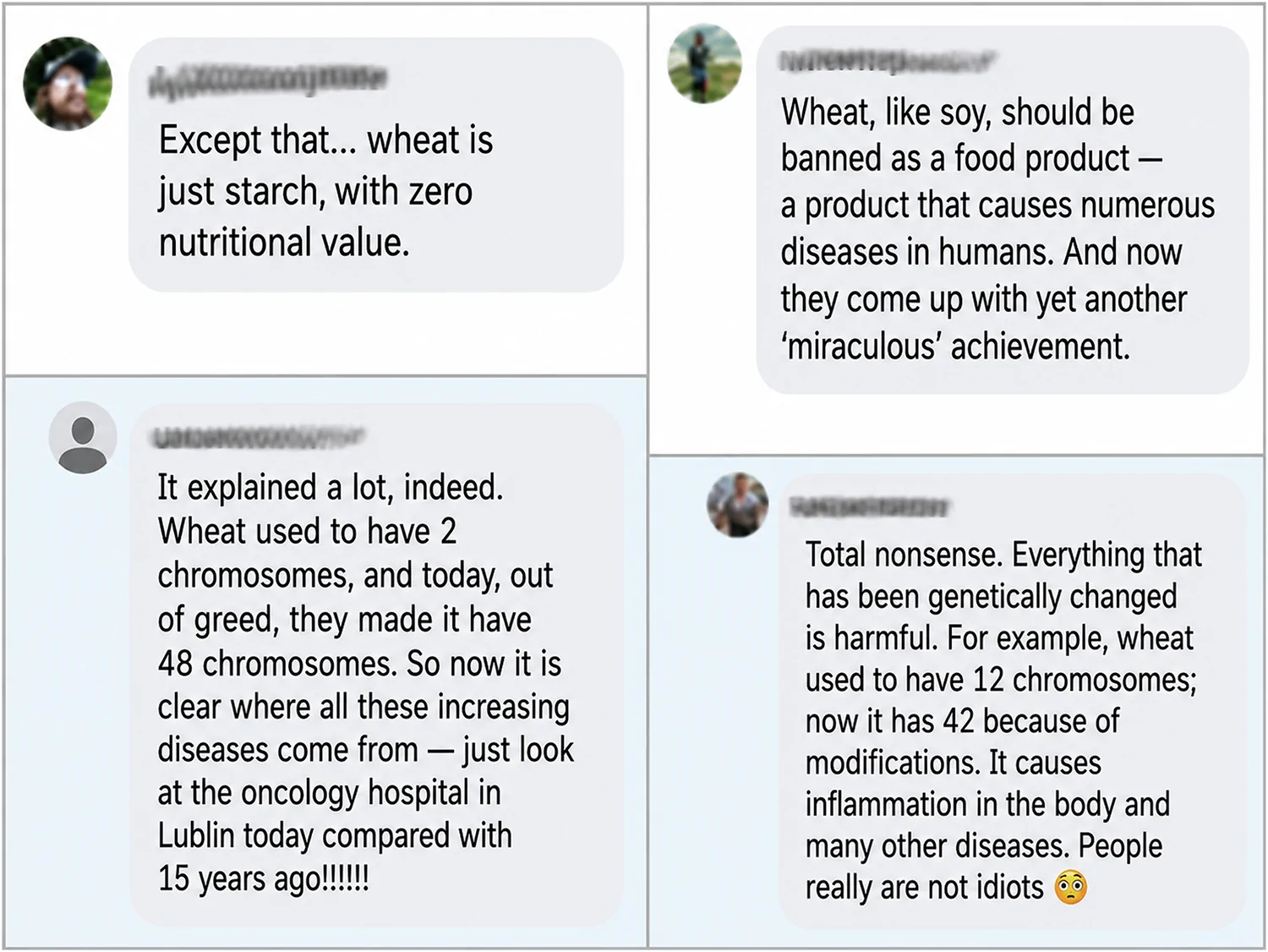
Anonymised examples of public misconceptions encountered during NGT outreach activities. Comments were translated from Polish; minor grammatical adjustments were made for clarity while preserving the original meaning and factual inaccuracies. The examples are illustrative, not a quantitative assessment of public opinion, and show how scientific-sounding terminology and numerical claims can strengthen biologically inaccurate messages in social media environments.

In addition, the—deliberately false—use of specific biological terms or numerical values in anonymous statements made them more believable and had a stronger impact on unsuspecting readers—even when those statements were clearly incorrect or outright bollocks (Fig. 1). Although anyone with some basic understanding of biology immediately understands that the commenter is either lying or has no clue of the subject, lay persons will still believe those ‘alternative facts’ and may repeat them in other discussions.

With rapidly developing AI tools, we observe another worrying trend: these tools make it even easier to fabricate and spread disinformation, instead of being used for fact-checking. The ability to have free access to most of the knowledge generated by humanity with just a few clicks does not promote critical analysis of information coming from uncertain sources. On the contrary, it is used to generate false information based on the selective interpretation of scientific reports or fabricated sources pretending to be scientific articles.

The ability of having free access to most of the knowledge generated by humanity with just a few clicks, does not promote critical analysis of information coming from uncertain sources.

## Algorithms, AI and the architecture of scientific communication

Science is not in a losing position though. The scientific community has the knowledge, institutions and technical resources to counter this worrying trend. However, it is not enough to simply produce more content or to expect audiences to find reliable information on their own among thousands of competing messages. We need an easily usable mechanism that allows people to find scientifically validated information and to distinguish evidence-based material from content that only imitates the language of science.

The solution should not be yet another website or social media platform, but a professionally managed repository, created by scientists, of verified scientific and educational materials, provisionally called “Wikipedia 2.0”. It should not compete with YouTube, TikTok, Instagram or traditional media. Instead, it would provide a universal resource for teachers, university lecturers, journalists, public institutions, science communicators and the public at large. A visible and crucial element of the system would be a quality mark, such as #VerifiedByScience, indicating that the content has been checked for scientific accuracy. Users should be able to easily identify the sources on which the material is based, who prepared it, who approved its final version and when it was updated.

## A knowledge pyramid

**Fig. 2:**
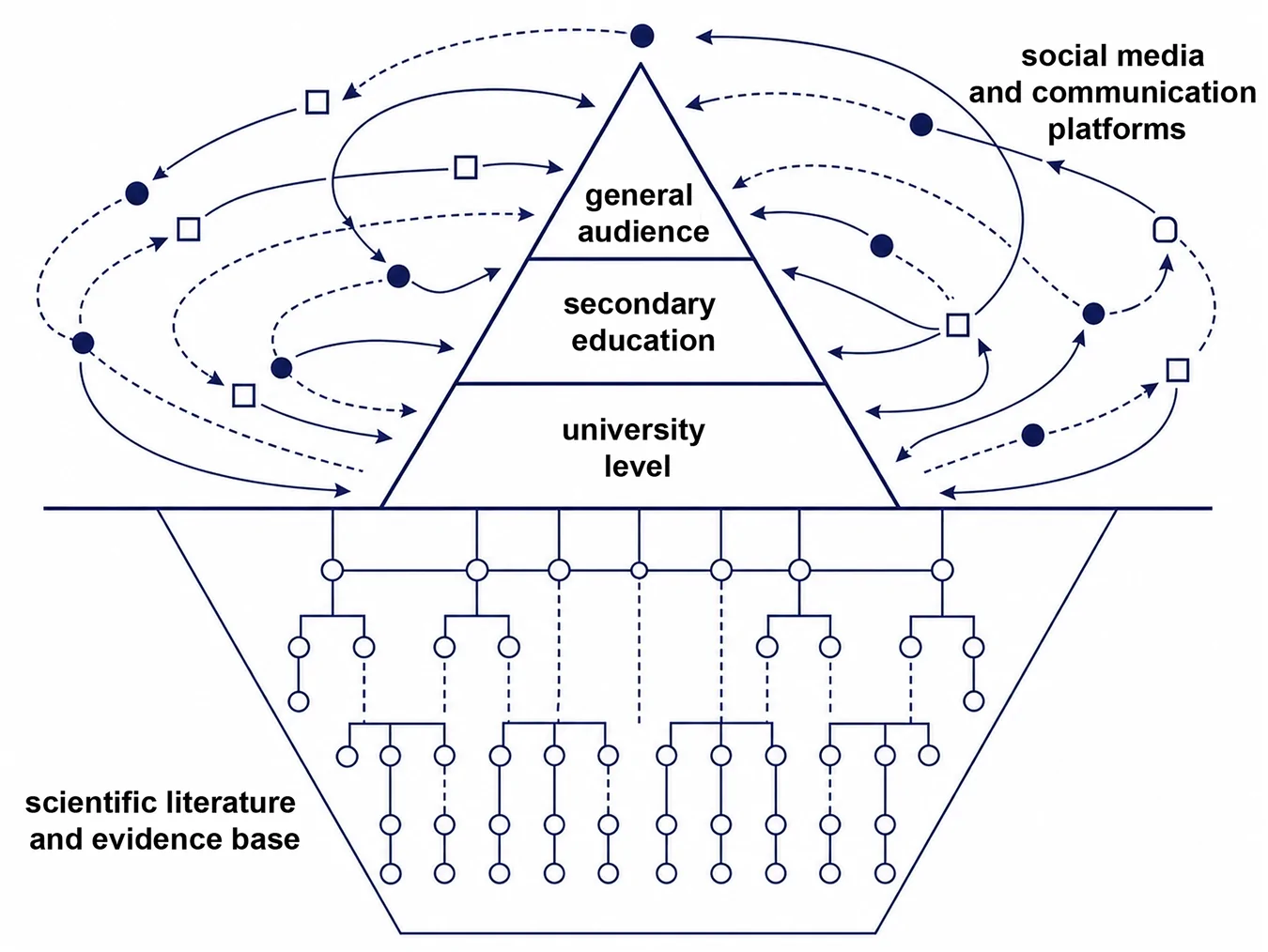
A multi-tiered ecosystem for scientific communication. Peer-reviewed literature and empirical data form the foundational evidence base, which supports successive layers of knowledge translation for academic, secondary-education and general-public audiences. The surrounding media ecosystem represents AI-assisted dissemination channels that adapt verified scientific content into accessible formats for social media without detaching it from its evidence base.

Scientific publications should form the foundation of the repository, but their results cannot be presented without the background knowledge needed to understand them. Every important discovery can become the starting point for a network of related educational materials. For example, when describing research on the possibility of silencing the third copy of human chromosome 21 (Lian et al, 2026), it is also possible to explain: what a chromosome and a genome are, why an additional copy of a chromosome causes Down syndrome and what lyonisation is. The same discovery can become the basis for a knowledge pyramid: specific content to address different groups. This would include a simple explanation for primary-school students, more detailed material for secondary-school students and an in-depth description for university students. Scientific publications and data form its foundation, followed by academic and educational materials, while short content for the general public is at the top (Fig. 2). A user may begin with a simple graphic or video and then move towards increasingly detailed levels of knowledge. The repository would therefore not be a collection of separate posts, but an organised network of content with different entry levels.

This knowledge source would not replace the educational content already available on other platforms. These materials would continue to serve their audiences independently. The specific role would be to provide a separate, evidence-based resource created and validated by identifiable members of the scientific community. Educators, journalists and online creators could freely use this resource when preparing their own content. Its added value would therefore not lie in replacing existing science communication, but in providing it with reliable and transparent information.

## Building recognition over many years

The repository would of course not immediately become a globally recognised resource. The #VerifiedByScience brand would have to be built consistently over many years, with schools and universities playing a particularly important role. If labelled materials were regularly used during lessons, lectures and online courses, successive generations of students would learn to see the repository as a common and reliable place to verify information. Not every user needs to analyse the scientific literature independently. It is enough that they recognise the label, know where to find the relevant material and share it in a discussion. In online communication, one person who posts a reliable explanation under a false comment may reach many undecided readers who are following the discussion. The repository would therefore provide not only knowledge, but also ready-to-use tools for spreading it further.

The repository would provide not only knowledge, but also ready-to-use tools for spreading it further.

During the initial stage, promotional campaigns would need to involve creators who are well known among young people in order to achieve wider use by the public. Influencers would only be responsible for presenting the repository in a way that is natural for a specific platform and for directing potential users towards it. Recognition could also be strengthened by local media. A short graphic or a 30-second animation used in a news programme, radio broadcast or online news portal could state that it came from a #VerifiedByScience source. The regular presence of the same label in schools, media, museums, public institutions and social media would gradually create a shared point of reference.

## Responding to current events

The repository should systematically build a permanent collection of content and then use current events to advertise it. Similar to the Science Media Centres in various countries, it could quickly react to unfolding news and provide journalists and the public with relevant and reliable information. If an accident occurs at a nuclear power plant, the repository could promote previously prepared content about atomic structure, radiation, radioactive decay and energy production. The release of a popular film about mutants could provide an opportunity to share material about genetic mutations, their causes and their biological consequences. An epidemic could increase the visibility of existing content about viruses, immunity and vaccination. AI could play an important role in tracking trends, detecting topics that are rapidly gaining attention and identifying which resources are particularly relevant at a given moment. It could also help refresh a description, prepare a new headline or adapt a graphic to a current context. However, the final decision about publication and its form should remain in human hands. This strategy would increase the chance that valuable material is noticed and shared because it appears exactly when audiences are interested in it. Reach would not be the goal in itself, but a tool for building awareness of the repository and directing users towards the verified knowledge there.

The repository would not eliminate misinformation from social media and would have no control over false claims published on external platforms. Its role would be different: to provide ordinary users, teachers, journalists and science communicators with reliable, accessible materials that they could use when responding to misinformation. In many online discussions, a single user sharing a clear and recognisable #VerifiedByScience resource may provide an important reference point for other readers following the exchange.

## Validation, AI and responsibility

The credibility of the repository must be paramount and based on a transparent quality-control process. Every material should undergo scientific, educational, editorial and visual validation. Scientists would check whether it reflects the current state of knowledge, teachers and education specialists would assess whether it is appropriate for the intended audience, editors would ensure clarity, and visual communication specialists would evaluate how processes and data are presented.

AI could significantly reduce the costs of translation, prepare different language versions and subtitles, and adapt existing material. However, every final version should be checked by a scientist who is fluent in the target language and familiar with the local terminology. AI would therefore support the scaling of the project, but final approval should remain a human responsibility.

Unlike Wikipedia, anonymous users would not be allowed to create, approve or modify validated content. These actions would be restricted to members of the scientific community, identifiable by their names, affiliations and ORCIDs. This would ensure both transparency and assure users that the content had been prepared and approved by members of the scientific community. At the same time, participation in the project should become a visible element of a researcher’s professional activity. Authorship or review of materials could be recorded in an ORCID profile, strengthen the expert’s public recognition and be considered by universities or research-funding institutions. Every material should also include a review date, version history, sources and a procedure for reporting errors.

## Governance and funding

A project of this scale cannot rely on the voluntary work of scientists. It would require a professional team, including positions dedicated to coordination, editing, visual production, cooperation with experts, management of language versions and promotion.

Scientific publishers and learned societies would be natural partners. Publishers have editorial infrastructure, experience in content management and direct contact with authors and reviewers. Learned societies provide networks of experts in individual disciplines. However, no publisher or single institution could or should control the repository. The optimal solution would be a consortium of multiple organisations with transparent governance rules. Funding could come from publishers, governments, research-funding agencies, the EU, international organisations and foundations involved in education and countering misinformation. These funds would support a small permanent professional team in the pilot phase and the basic IT infrastructure. Compared with the cost of conducting scientific research, this would be a relatively small investment but would drastically increase the social value of knowledge financed with public money.

Scientific content should be supervised by openly identified thematic boards composed of representatives from different institutions and countries. Technical administrators should not be allowed to make independent decisions on scientific matters. Fixed terms of office, disclosed conflicts of interest, version histories and formal reassessment procedures would limit the risk of control being taken over by anonymous administrators or interest groups.

## From a pilot project to shared infrastructure

The repository could begin as a limited pilot project covering several disciplines, institutions and languages. The first objective should be to prepare an organised collection of basic materials, test the validation system and determine whether schools, media organisations and online creators actually use the resources. Evaluation should include not only the number of views, but also recognition of the quality mark, understanding of the content and the willingness of users to reuse the material. If the pilot proves to be effective, the repository could gradually be expanded to include more disciplines and languages.

It would not eliminate misinformation or replace education. However, it could create a shared point of reference that is currently missing: a professionally curated, transparent and easily reusable resource of scientific knowledge. The scientific community already has most of the necessary elements available: knowledge, experts, publishers, learned societies, educational institutions and AI tools. What is mainly missing is a common standard, stable funding and the decision to treat science communication as social infrastructure rather than as an additional activity carried out by individual enthusiasts.

What is mainly missing is a common standard, stable funding and the decision to treat science communication as social infrastructure rather than as an additional activity carried out by individual enthusiasts.

## Supplementary information

Peer Review File
