# Supplementary material for: When expertise became just another opinion: Rebuilding trust through a transparent infrastructure for verified scientific knowledge
Source: EMBO Rep. 2026 Aug 12;27(17):4945–9. doi: 10.1038/s44319-026-00899-x (PMC13554085; doi:10.1038/s44319-026-00899-x)
Supplement: Supplementary file 1 [file 44319_2026_899_MOESM1_ESM.pdf]

EMBO reports

When expertise became just another opinion

Marek Marzec

Corresponding author: Marek Marzec (marek.marzec@us.edu.pl)

Review Timeline:

Submission Date:	31st Jul 26
Accepted:	4th Aug 26

Editor: Holger Breithaupt

Transaction Report:

This article was editorially reviewed.
